# Perceived causal relations between anxiety, posttraumatic stress and depression: extension to moderation, mediation, and network analysis

**DOI:** 10.3402/ejpt.v4i0.20656

**Published:** 2013-08-30

**Authors:** Paul A. Frewen, Verena D. Schmittmann, Laura F. Bringmann, Denny Borsboom

**Affiliations:** 1 Department of Psychiatry and Psychology, Graduate Program in Neuroscience, Western University Canada, London, Ontario, Canada; 2 Department of Psychological Methods, University of Amsterdam, Amsterdam, The Netherlands; 3 Department of Quantitative Psychology and Individual Differences, University of Leuven, Leuven, Belgium

**Keywords:** Perceived causal relations, comorbidity, assessment, posttraumatic stress disorder (PTSD), depression, anxiety

## Abstract

**Background:**

Previous research demonstrates that posttraumatic memory reexperiencing, depression, anxiety, and guilt-shame are frequently co-occurring problems that may be causally related.

**Objectives:**

The present study utilized Perceived Causal Relations (PCR) scaling in order to assess participants’ own attributions concerning whether and to what degree these co-occurring problems may be causally interrelated.

**Methods:**

288 young adults rated the frequency and respective PCR scores associating their symptoms of posttraumatic reexperiencing, depression, anxiety, and guilt-shame.

**Results:**

PCR scores were found to moderate associations between the frequency of posttraumatic memory reexperiencing, depression, anxiety, and guilt-shame. Network analyses showed that the number of feedback loops between PCR scores was positively associated with symptom frequencies.

**Conclusion:**

Results tentatively support the interpretation of PCR scores as moderators of the association between different psychological problems, and lend support to the hypothesis that increased symptom frequencies are observed in the presence of an increased number of causal feedback loops between symptoms. Additionally, a perceived causal role for the reexperiencing of traumatic memories in exacerbating emotional disturbance was identified.

In the science and practice of clinical psychology and psychiatry, questions concerning the causality of one clinical problem for another are commonplace. For example, in a depressed person with relationship problems, clinicians may hypothesize an individual's interpersonal problems as a significant cause and/or outcome of his or her depression. Indeed, clinical problems often present within complex causal chains, such as in the case of interpersonal problems (e.g., social rejection) initially causing a depressive episode that in turn causes further social rejection (e.g., reviews by Joiner, 2000; Liu & Alloy, 2010; Monroe & Harkness, 2005). Nevertheless, bidirectional causal pathways can vary in dominance (Kraemer, Stice, Kazdin, Offord, & Kupfer, 2001) such as when an individual's relational problems strongly cause his or her depression, but his or her depression only moderately causes further interpersonal problems.

Individuals themselves perceive their behavior, life situations, and (sub-) clinical psychological and physical symptoms as causally related (Frewen, Allen, Lanius, & Neufeld, 2012). Although such causal attributions may deviate from actual causal relations, they are of interest to measure in and of their own right, revealing how participants’ think about themselves and their problems (Frewen et al., 2012). In the area of physical illnesses, patients’ own causal attributions have been shown to be predictive of health behavior, compliance, and recurrence, affecting overall psychological adjustment (see Brogan & Hevey, 2009, for an overview). Unfortunately, mainstream clinical assessment methods cannot be used in order to assess the causal relatedness often perceived to exist between co-occurring psychological problems. In fact, current approaches effectively assess clinical problems as if they might exist in isolation. To illustrate, clinicians may assess a client's depression with one measure, and his or her interpersonal problems with another, but they have no psychometrically validated methods available to them in order to assess whether a client perceives the two problems as related, and if so to what extent and how. Accordingly, researchers have argued that developing an assessment methodology aimed at elucidating the causal significance of co-occurring psychological problems at the idiographic case level could significantly aid clinical case conceptualization (Frewen et al. 2012; Haynes, Mumma, & Pinson, 2009). For example, assessing the perceived causal pathways associating a person's interpersonal problems with his or her depression could inform the question as to whether psychological treatment should address the individual's interpersonal problems before, after, or simultaneously to addressing his or her depression itself.

In this article, we investigate the coherence of perceived causal structures between psychological problems, as reported by participants, at the level of psychological disorders and at the symptom level. We first introduce the assessment of the causal structure, and then derive our hypotheses concerning coherence of the causal structure.

## Perceived causal relation scaling

We recently developed a psychometric methodology for assessing participants’ own attributions concerning possible causal interrelationships associating their co-occurring presenting problems (Frewen et al., 2012). Our methodology, which we titled “Perceived Causal Relationship (PCR) scaling,” requires participants to rate, with regard to all surveyed clinical problems present, the degree to which they attribute each problem as the cause of all other individual problems they endorse. Stated simply, if two variables x and y are present, PCR scaling requires participants to answer two “causal association” questions, specifically: (1) “How much do you think your problems with [x] cause your problems with [y]?” and (2) “How much do you think your problems with [y] cause your problems with [x]?.” As used herein, Likert-scale scores provided as answers to each question are denoted PCR_*x*→*y*_ and PCR_*y*→*x*_, respectively. Accordingly, referring to *x*, PCR_*x*→*y*_ indicates the perceived *causal* association of *x* for *y*, whereas PCR_*y*→*x*_ indicates the perceived *effect* association of *x* for *y*. Answers to preceding questions concerning the simple frequency of *x* and *y* may be denoted by *x*_FREQ_ and *y*_FREQ_. Being that what clinical problems are endorsed (i.e., *x*_FREQ_ and *y*_FREQ_ scores) vary across individuals, so will the causal association questions that are indicated for follow-up; although this makes the procedure cumbersome to administer by interview or paper-and-pencil survey, it is easily implemented via computerized adaptive testing procedures (Forbey & Ben-Porath, 2007; Garb, 2007).

Frewen et al. (2012) identified non-zero PCR between depression and each of intrusive reexperiencing and anxiety, a result consistent with perceived bidirectional causality. However, participants attributed their intrusive reexperiencing and anxiety as stronger causes of their depression than vice versa, indicative of differential dominance (Kraemer et al., 2001). While the assessment of PCR has been established, the coherence of the causal structure, that is, whether and how PCR scores and symptom frequency ratings are related, remained unknown. For instance, if symptom A actually causes symptom B and vice versa (i.e., a feedback loop), we expect stronger symptom frequencies of both symptoms as an outcome than if symptom A causes symptom B, but B does not cause A. Such coherence of the perceived causal structure is important to establish, as its absence could point to an omitted relevant cause C (influencing both A and B), and/or to differences between perceived and actual causal relations.

## PCR scaling and moderator analyses

**Fig. 1:**
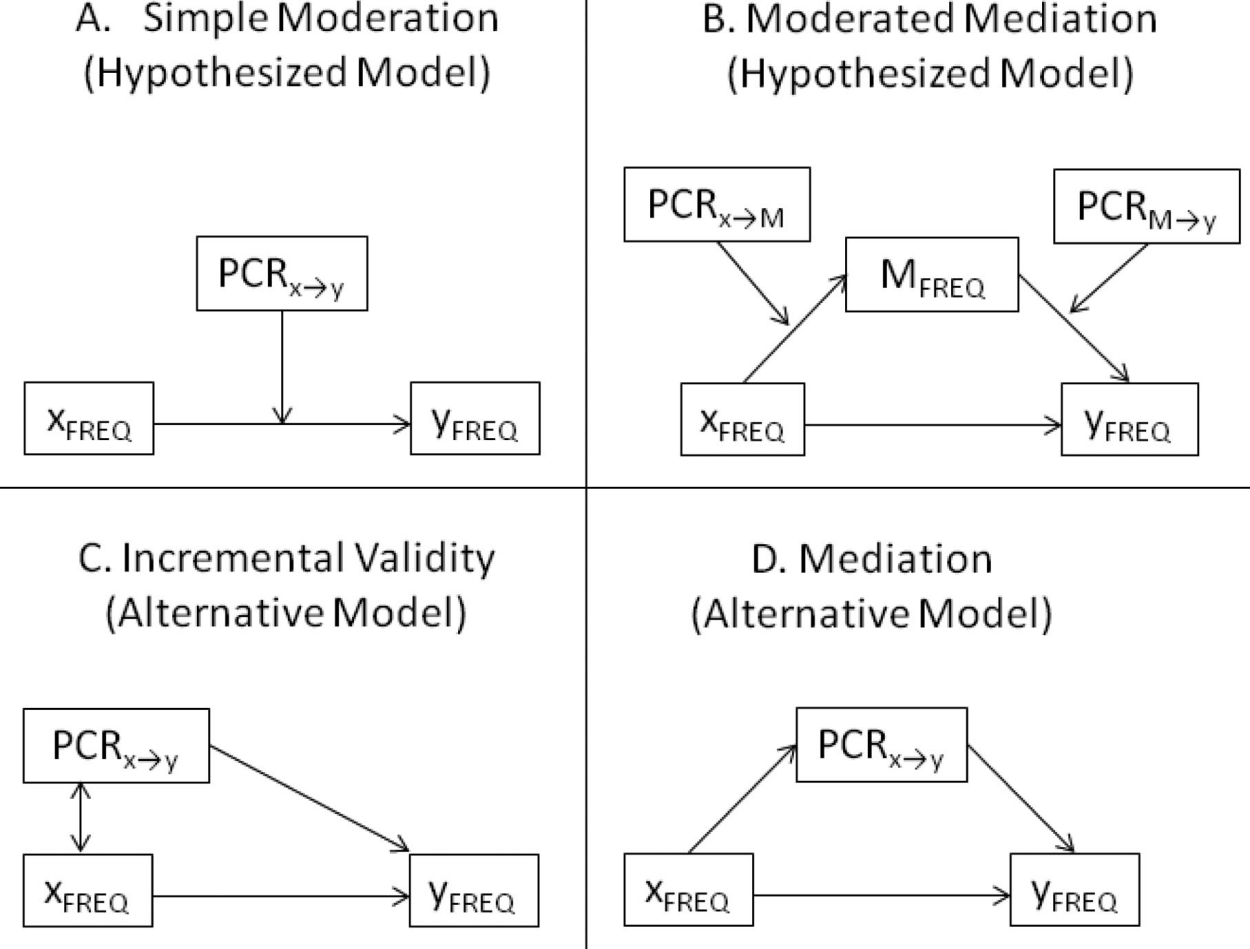
Hypothesized moderator vs. alternative incremental validity and mediation models of the association between PCR scores and symptom frequency scores.

The first objective of this study was to evaluate the hypothesis that PCR scores moderate the association between relevant symptom frequency scores (i.e., we hypothesized that PCR_*x*→*y*_ would moderate the concurrent prediction of *y*_FREQ_ by *x*_FREQ_; see Fig. 1A). Specifically, we reasoned that, if participants’ PCR_*x*→*y*_ scores in any way approximate the extent to which *x*_FREQ_ acts as a causal risk factor for *y*_FREQ_, higher PCR_*x*→*y*_ scores should be associated with stronger correlations between *x*_FREQ_ and *y*_FREQ_ (i.e., the correlation between *x*_FREQ_ and *y*_FREQ_ should vary as a positive function of PCR_*x*→*y*_ scores; Fig. 1A). For example, applying this logic to the case of reexperiencing, anxiety, and depressive symptomatology, our argument is that, if intrusive reexperiencing (REEXP_FREQ_) and anxiety symptoms (ANX_FREQ_) represent causal risk factors for depressive symptoms (DEP_FREQ_), and PCR_REEXP→DEP_ and PCR_ANX→DEP_ scores approximate the true degree to which REEXP_FREQ_ and ANX_FREQ_ are causally related to DEP_FREQ_ across persons, PCR_REEXP→DEP_ and PCR_ANX→DEP_ scores should positively predict the strength of the association between DEP_FREQ_ and each of REEXP_FREQ_ and ANX_FREQ_, respectively. In addition to simple moderation models (Fig. 1A) of the effects of PCR scores in moderating associations between reexperiencing, depression, and anxiety, we also tested a moderated mediation model (Muller, Judd, & Yzerbyt, 2005; Preacher, Rucker, & Hayes, 2007) wherein the mediator was represented by another symptom frequency score. In the moderated mediation model, the causal paths among symptom frequencies, as was the case in the simple moderation models, were hypothesized to be moderated by PCR scores (see Fig. 1B).

The moderated mediation analysis further evaluated the association between intrusive reexperiencing of traumatic events and depression (e.g., Brewin, Gregory, Lipton, & Burgess, 2010). Specifically, we evaluated whether reexperiencing of traumatic events partially mediated the robust association established between guilt–shame and depression (Kim, Thibodeau, & Jorgensen, 2011). We examined reexperiencing symptoms as a candidate mediator of the association between guilt–shame and depression because guilt–shame is a well-known correlate of posttraumatic stress (e.g., Andrews, Brewin, Rose, & Kirk, 2000; Brewin, Andrews, & Rose, 2000; Budden, 2009; Harman & Lee, 2010; Holmes, Grey, & Young, 2005; Lee, Scragg, & Turner, 2001; Leskala, Dieperink, & Thuras, 2002; Wilson, Droždek, & Turkovic, 2006; Wong & Cook, 1992) and recent research establishes the occurrence and characteristics of traumatic memories as strongly predictive of experiences of guilt and shame that, in turn, are predictive of depressive symptoms (Matos & Pinto-Gouveia, 2010; Matos, Pinto-Gouveia, & Costa, 2011; Matos, Pinto-Gouveia, & Duarte, 2012; Pinto-Gouveia & Matos, 2011; Robinaugh & McNally, 2010). Researchers have therefore argued that posttraumatic reexperiencing symptoms may be generated and maintained not only by fear but also by shame, in turn sometimes engendering an especially dark and depressive narrative of the self as broken, defective, defeated, and, depending on the social and moral relevance of the traumatic event, defiled, dirty, and even repulsive (e.g., Frewen et al., 2011; Litz et al., 2009; Matos et al., 2012). As such, our moderated mediation analysis sought to answer Kim et al.'s call for further research examining the mediating pathways through which guilt and shame are associated with depression (Kim et al., 2011). Following Matos et al. (2012), we hypothesized that guilt and shame experiences may be highly central to the self-schema of certain traumatized persons, in turn frequently priming the intrusive recall of traumatic memories and engendering depressive symptoms. We also examined whether participants’ own attributions concerning PCR associating their guilt–shame with their reexperiencing symptoms, and in turn their reexperiencing symptoms with their depression, partially explained variation in the degree to which reexperiencing mediated the association between guilt–shame and depressive symptoms (see Fig. 1B).

**Fig. 2:**
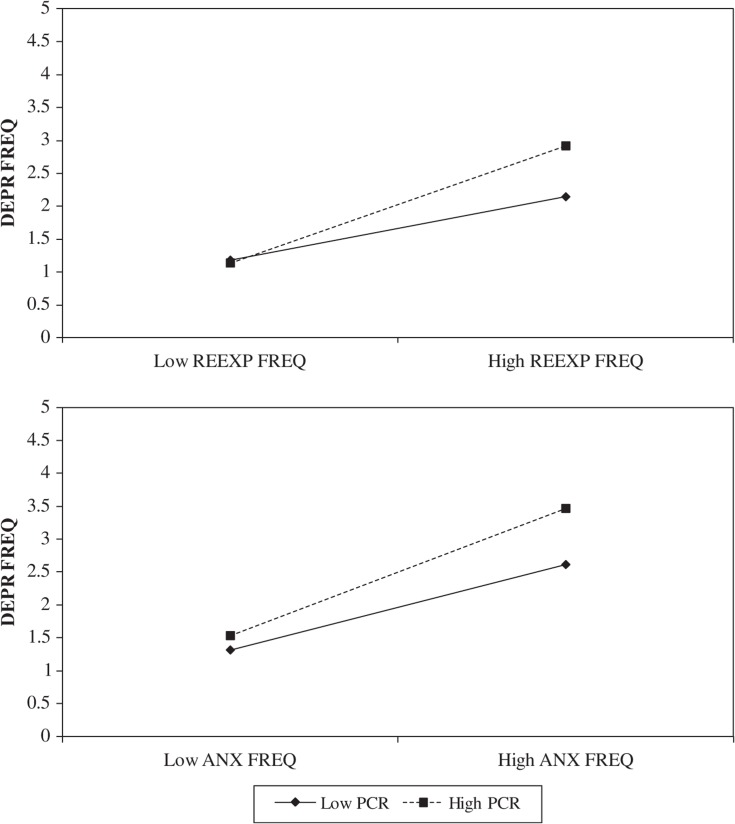
Simple slopes between depression symptom frequency and each of anxiety and reexperiencing symptom frequency at low versus high PCR scores.

While preferring the conceptualization of PCR scores as moderators, we contrasted our moderation hypothesis with two alternatives, those conceptualizing PCR_*x*→*y*_ scores as (1) either overlapping or independent risk factors (Fig. 1C), or (2) mediators (Fig. 1D), of the association between *x*_FREQ_ and *y*_FREQ_ (Hayes, 2009, 2013; Kraemer et al., 2001). Given previous findings of only small or null associations between PCR scores and corresponding symptom frequency scores (Frewen et al., 2012), conceptualizing PCR_*x*→*y*_ scores as either overlapping or independent risk factors was not a preferred hypothesis. Moreover, given that we conceive of PCR_*x*→*y*_ scores as indicative of the *strength* of the perceived causal association between *x*_FREQ_ and *y*_FREQ_, rather than as providing an explanatory *mechanism* through which *x*_FREQ_ causes *y*_FREQ_, we neither preferred the hypothesis of PCR_*x*→*y*_ as a mediator of the association between *x*_FREQ_ and *y*_FREQ_. To summarize, the top quadrants of the figure (Fig. 1A and 1B) illustrate the hypothesized moderator models associating PCR ratings with symptom frequency scores, whereas the bottom quadrants illustrate the alternate hypotheses, whether conceptualizing PCR ratings as either overlapping or independent risk factors (Fig. 1C, bottom left) or mediators (Fig. 1D, bottom right).

## PCR scaling and network analyses

A second objective of this study was to further explore the internal coherence of the whole perceived causal structure, that is, including all symptoms, utilizing analytic methods associated with Borsboom et al.'s *network approach* to psychometric theory and comorbidity studies (Borsboom, 2008; Borsboom & Cramer, 2013; Borsboom, Cramer, Schmittmann, Epskamp, & Waldorp, 2011; Cramer, Waldrop, Van der Maas, & Borsboom, 2010; Schmittmann et al., 2011). The network theory models causal interrelationships between measured variables (e.g., clinical symptoms/problems) as an explanation of the co-occurrence or correlation between latent factors (e.g., comorbid disorders or syndromes). Although our previous study identified PCR across numerous clinical problems, only *direct* causal associations have so far been examined (e.g., *x*→*y*), as opposed to *indirect* causal associations (e.g., *x*→*z* as a function of *x*→*y* and *y*→*z*). In order to examine multi-problem causal pathways within PCR scores in a way that is in better keeping with the presumed complex causal chains more often linking comorbid clinical problems, this study applied PCR scaling to *network analyses* (e.g., Cramer et al., 2010; Schmittmann et al., 2011). The measurement objectives of PCR scaling emulate the network theory of psychopathology through their examination of symptom-to-symptom bidirectional causal relationships as a theoretical account of comorbidity at the diagnostic level, such as between reexperiencing, anxiety, and depression. As one measure of indirect causal associations, we calculated the *betweenness centrality* of each symptom from the network of average PCR scores. A symptom's betweenness centrality measures the extent to which the symptom lies on the shortest causal paths between two other symptoms (Opsahl, Agneessens, & Skvoretz, 2010). We hypothesized that higher symptom frequency ratings would be associated with a larger number of causal feedback loops between symptoms (e.g., intrusive reexperiencing and anxiety symptom frequencies may be increased in the presence of one or more feedback loops between them).

## Method

### Participants

A total of 288 undergraduate students at Western University in London, Ontario, Canada participated in this study for partial course credit. Sample characteristics closely matched a previous study (Frewen et al., 2012) as follows: most participants were women (*n=* 206, 72%) and in their late adolescence, 91% being between 19 and 23 years of age (M=21.73, SD=3.60, range: 19–48). Marital status was predominantly single (88%, *n*=253). Ethnic status was assessed in 96 participants; of these 68 (71%) described themselves as of European–Caucasian (EC) descent. Forty-six participants (16%) answered in the affirmative when asked if they had “ever been diagnosed with a psychiatric disorder by a physician or psychologist.”

#### PCR scaling

PCR scaling was conducted as in a previous study (Frewen et al., 2012). Forty items were previously developed by Frewen et al. (2012)) in order to measure all symptoms of a major depressive episode (MDE; 10 items), all but one symptom of DSM-IV posttraumatic stress disorder (PTSD) (16 items; amnesia for traumatic events was excluded due to poor factor representation in previous research, for example, King, Leskin, King, & Weathers, 1998), symptoms of anxiety disorders (four items measuring: (1) panic attacks, (2) generalized worry, (3) social anxiety, and (4) agoraphobia), and additional single items intended to screen for other psychological difficulties that often co-occur with MDE, PTSD, and anxiety disorders (e.g., hypomania, substance-abuse, dissociation, self-harm, sexual problems, pain problems, social and occupational impairment). A single item was also used in order to assess experiences of guilt and/or shame as follows: “Extreme guilt and/or shame about things that you have done, failed to do, or have happened to you (feeling at fault, to blame, having a strong sense of shame).” Face validity relative to definitions for the same symptoms as taken verbatim from the DSM-IV-TR (American Psychiatric Association, 2000) was confirmed in a previous pilot study (20 participants were required to match randomly sorted PCR symptom definitions to their DSM-IV-TR counterparts; mean hit rate was 84% [SD=14%]; Frewen et al., 2012).

Participants were first asked in regard to each item “How frequently have you experienced this problem in the past month?” and responded by clicking from a drop-down menu from one of eight response options ranging between “Not at all in the past month” and “Daily or almost daily for most of the day” and scored 0–7, respectively. Supporting the convergent validity of the items measuring MDE and PTSD, Frewen et al. (2012) demonstrated in an undergraduate sample (*n*=225) that endorsement of MDE items correlated *r*=0.77 with *Beck Depression Inventory – II* scores (Beck, Steer, & Brown, 1996) and endorsement of PTSD items correlated *r*=0.68 with *Posttraumatic Diagnostic Scale* scores (Foa, 1995).

All items that were reported present at least “Once in the past month” were then inserted into follow-up *causal association* questions. Participants were asked “How much do you think your problems with [inserting some ‘Symptom X’] CAUSE your problems with [inserting some ‘Symptom Y’]?” and, likewise, “How much do you think your problems with [inserting some ‘Symptom Y’] CAUSE your problems with [inserting some ‘Symptom X’]?” Thus, for any given item pairing, participants were asked the *causal association* question in each of its two permutations. Answers to the follow-up *causal association* questions were given via a drop-down menu with response options from 0–10, with 0, 5, and 10 denoting “Not at all,” “Moderately cause,” and “Strong cause,” respectively, and no additional item anchors.

Note that causal associations that are not rated as a result of either/both of the symptoms being reported absent in the last month are treated as missing variables. In other words, such variables are missing of necessity or “by design.” For ethical reasons, participants also had the opportunity to select “Skip this question” as a response option, with the computerized assessment procedure itself ensuring that a response option was provided to all questions. In this case, such values were missing “by intention” of the participant.

### Procedure

Participants signed up for the study online, and completed the PCR assessment on their own via the internet at a place of convenience to them; they received a participation credit toward completion of an undergraduate psychology course for doing so. An institutional ethics committee approved the study procedure.

### Statistical analysis

Statistical analysis was carried out on all available data without replacement; thus values missing either “by design” or “by intention” (see “Methods” section) were not differentiated or represented in the study results. We calculated *Mean Causal Association* and *Mean Effect Association* scores as outlined in Frewen et al. (2012). The *Mean Causal Association* score for any given item is the average *Causal Association* score the symptom receives across all other items rated when occupying “Symptom X” in the causal association question: “How much do you think your problems with [Symptom X] CAUSE your problems with [Symptom Y]?” In comparison, the *Mean Effect Association* score is the average *Causal Association* score when occupying “Symptom Y” in the same question. Such scores therefore represent the extent to which individual items are attributed, on average, as the cause versus effect (outcome), respectively, of all other items rated present. Paired differences between mean causal and effect association scores were accepted as statistically significant only after the Holm-Bonferroni (“Sequential-Bonferroni”) correction for multiple comparisons.

Mean causal and effect association scores, however, need not average across all items but instead can be calculated across item subsets focused on particular contents. Consistent with the latter approach, and further following Frewen et al. (2012), we also calculated mean causal and effect association scores particular to each of: (1) the four anxiety items (ANX; items 1–4), (2) the 10 depression items (DEP; items 12, 13, 18, 24, 27–30, 32, 33), and (3) the five PTSD reexperiencing (DSM-IV PTSD criteria “B”) items (REEXP; items 5–9). These variables were then utilized in tests of PCR scores as incremental predictors, mediators, and moderators of associations between symptom frequency scores (see Fig. 1).

The PROCESS macro (Hayes, 2013) implemented in SPSS 20 was utilized to estimate the mediation and moderation models (Fig. 1A, B, and D were tested with models 1, 21, and 4 in PROCESS, respectively). PROCESS utilizes a boot-strapping approach (10,000 samples as tested herein) to evaluate the 95% confidence limits of the size of particular model-specified indirect effects. The boot-strapping approach, such as that implemented in PROCESS, is an increasingly favored method to testing mediation models within the literature relative to the highly familiar Baron and Kenny (1986) *causal steps* approach on both logical grounds (e.g., Hayes, 2009) as well as for its increased sensitivity to detecting true indirect effects (e.g., MacKinnon, Lockwood, Hoffman, West, & Sheets, 2002). In comparison, the model implicating PCR scores as simple incremental predictors (as in Fig. 1C) was tested via standard SPSS linear regression. To address risk for type-1 error, statistical significance was accepted only after Bonferroni correction, with analyses of anxiety and reexperiencing treated as different families of tests. Accordingly, as three models were tested (incremental prediction, mediation, and moderation), *α* was set at *p-critical*=0.05/3=0.017 (98.33% confidence intervals [CI] are thus reported as the Bonferroni-corrected 95% CI).

We also generated a directed network of PCR scores at the symptom level using the R-package qgraph (Epskamp, Cramer, Waldorp, Schmittmann, & Borsboom, 2012). In this network, nodes represent symptoms and PCR scores are represented by edges between the nodes. The direction of the edges indicates the direction of the perceived causal effect, and the thickness indicates its strength.

To examine clustering and centrality in the symptom space, we employed an algorithm that minimizes edge crossing and takes symmetry into account leading more strongly connected sets of symptoms to cluster closer together (Fruchterman & Reingold, 1991). Additionally, we explored which symptoms are most central or influential in the network. Since the edges between the symptoms differ in strength, we used the following three centrality measures for such weighted networks (Opsahl, Agneessens & Skvoretz, 2010): outdegree, indegree, and betweenness centrality. Outdegree reflects the sum of the weight of the arrows leaving a node, whereas indegree indicates the sum of the weight of the arrows arriving at a node. Out- and indegree may seem similar to mean causal and effect association scores, respectively, but are different measures. Mean causal association scores assess the average influence of a symptom on those symptoms to which it is connected, while outdegree assesses the global influence of a symptom on *all* other symptoms.^1^ In- and outdegree are very informative, as these measures take into account the *direct associations* between symptoms.

However, equally important are *indirect associations* that emerge from the overall structure of the network (e.g., Worrying indirectly causing Fatigue, via Sleeping problems). Betweenness centrality builds on direct *and* indirect associations, such that symptoms that funnel activation flow through the network stand out with high betweenness centrality (Opsahl, Agneessens, & Skvoretz, 2010).

These three centrality measures were statistically evaluated by bootstrapping. For this purpose, we obtained distributions of centrality measures from 1,000 networks that were created by randomly permuting the observed mean causal association scores. Against these sampled distributions, we evaluate the observed centrality measures.

Finally, we examined the coherence of the complete symptom networks. For this purpose, we identified the feedback loops in each participant's network of PCR scores above 4.5, using the R-package LoopAnalyst (Dinno, 2009). Feedback loops exist when symptoms form a loop, so that the output of a symptom also influences the input to that same symptom, either directly or indirectly. Considering computational feasibility, feedback loops involving no more than four different symptoms were calculated. We used Spearman's correlation to assess magnitude and significance of the association between the number of feedback loops and the symptom frequency sum scores. As the maximum possible number of feedback loops depends on a network's number of symptoms and number of edges, we recalculated Spearman's rho, partialling out these two variables.

## Results

Most participants answered all questions, with only 2% of all frequency ratings and <1% of all PCR ratings missing “by intention” (i.e., participant chose not to answer the questions; see “Methods” section). Such values were not replaced, and all available data was submitted to statistical analysis.

### Descriptive statistics

Across participants, posttraumatic stress, depressive, anxiety, and other psychological symptoms were endorsed with varying frequency (see Table 1).

**Table 1:** PCR descriptive statistics and comparison of the mean cause versus effect status of symptoms

No.	Symptom (SCALE, abbreviation as presented in Fig. 4)	Frequency (F) M	Frequency (F) SD	Cause (C) M	Cause (C) SD	Effect (E) M	Effect (E) SD	*r*_EC_	*t*_EC_	*df*_EC_	*p_(tEC)_*	*d′*_EC_
1	Panic attacks (ANX, PANC)	0.85	1.20	2.11	2.03	2.22	1.90	0.65	−0.78	130	0.44	–
2	Anxious worrying (ANX, WRRY)	1.42	1.61	3.98	2.18	2.91	2.02	0.64	7.92	173	<0.001	0.61
3	Social anxiety (ANX, SCAX)	0.79	1.32	2.93	2.38	2.82	2.02	0.72	0.71	110	0.48	–
4	Agoraphobic behavior (ANX, AGOR)	0.37	1.08	2.37	2.40	2.65	2.13	0.71	−1.08	44	0.29	–
5	Intrusive memories of a traumatic event (PTSD, MEMT)	0.59	1.19	3.28	2.63	2.40	2.13	0.80	5.20	83	<0.001	0.57
6	Dreams/nightmares about a traumatic event (PTSD, DRM)	0.33	0.82	2.06	2.28	2.02	1.72	0.81	0.21	59	0.83	–
7	Emotional upset at reminder of a traumatic event (PTSD, EMOT)	0.59	1.18	3.56	2.62	2.73	2.02	0.83	5.10	81	<0.001	0.56
8	Physiological reaction at reminder of a traumatic event (PTSD, PHYS)	0.43	1.06	2.95	2.65	2.55	2.09	0.87	2.35	60	0.02	0.30
9	Flashbacks of a Traumatic Event (PTSD, FLSH)	0.26	0.84	3.79	2.86	2.86	2.16	0.81	3.45	37	0.001	0.56
10	Avoidance of thoughts/feelings about a traumatic event (PTSD, AVTH)	0.41	1.02	2.63	2.51	2.48	2.18	0.89	1.01	62	0.32	–
11	Avoidance of reminders of a traumatic event (PTSD, AVAC)	0.38	1.13	2.88	2.60	2.90	2.38	0.85	−0.09	46	0.93	–
12	Loss of interest (PTSD & MDD, LSIN)	0.89	1.43	2.72	2.24	2.91	2.13	0.77	−1.43	123	0.16	–
13	Depressed mood (MDD, DPRM)	1.24	1.47	3.86	2.51	3.33	2.22	0.77	4.29	177	<0.001	0.32
14	Feeling distant or cut off from others (PTSD, DIST)	1.22	1.58	2.96	2.0	3.04	2.14	0.84	−0.69	156	0.49	–
15	Emotional numbness (PTSD, NUMB)	0.67	1.24	2.87	2.41	3.03	2.20	0.85	−1.24	91	0.22	–
16	Sense of foreshortened future &/or loss of core life goals (PTSD, FRZN)	0.93	1.43	3.04	2.42	2.68	2.09	0.78	2.59	120	0.01	.24
17	Irritability/anger (PTSD, IRRI)	1.05	1.35	2.37	2.18	2.86	2.00	0.85	−5.19	143	<0.001	0.43
18	Thinking/concentration problems (PTSD & MDD, DCNC)	1.61	1.75	2.39	2.24	3.16	2.16	0.74	−6.54	184	<0.001	0.48
19	Hypervigilance (PTSD, HVGL)	0.85	1.44	2.06	2.31	1.90	1.90	0.76	1.08	110	0.28	–
20	Strong startle reactions (PTSD, STRT)	0.85	1.42	1.41	1.72	1.76	1.77	0.84	−3.71	111	<0.001	0.35
21	Derealization (DISSOC, DREA)	0.50	1.10	2.04	2.39	1.98	2.07	0.88	0.42	74	0.68	–
22	Depersonalization (DISSOC, DPRS)	0.47	1.05	2.06	2.12	2.23	1.98	0.76	−1.01	71	0.32	–
23	Identity confusion (DISSOC, IDCF)	0.59	1.20	2.55	2.43	2.47	2.07	0.90	0.68	82	0.50	–
24	Feeling worthless (MDD, WRTL)	0.80	1.45	3.39	2.47	2.95	1.88	0.87	3.62	103	<0.001	0.35
25	Guilt and/or shame (OTHER, SHME)	1.11	1.54	2.96	2.27	2.68	1.83	0.77	2.31	138	0.02	0.20
26	Self-harming behavior (OTHER, SHRM)	0.16	0.64	1.76	1.87	1.92	1.96	0.76	−0.61	25	0.55	–
27	Suicidal thinking/behavior (MDD, SUIC)	0.28	0.80	2.44	2.36	2.69	1.89	0.71	−0.90	37	0.37	–
28	Psychomotor agitation (MDD, AGIT)	0.79	1.41	2.25	2.20	2.58	1.94	0.78	−2.39	102	0.02	.24
29	Psychomotor slowing (MDD, SLOW)	0.34	0.89	2.16	2.16	2.57	1.92	0.86	−2.82	56	0.007	0.37
30	Energy loss/fatigue (MDD, FTIG)	1.35	1.71	2.58	2.12	2.76	1.96	0.79	−1.65	160	0.10	–
31	Hypomania (OTHER, HPOM)	0.24	0.60	1.76	2.22	1.68	0.80	0.89	0.52	46	0.61	–
32	Sleeping problems (PTSD & MDD, SLP)	1.52	1.80	2.72	2.17	2.98	2.10	0.75	−2.13	153	0.04	.17
33	Eating problems (MDD, EAT)	1.23	1.68	1.96	2.11	2.61	2.07	0.72	−4.93	136	<0.001	0.42
34	Sexual problems (OTHER, SEX)	0.37	1.01	1.55	1.84	2.26	1.89	0.66	−3.29	49	0.002	0.47
35	Pain problems (OTHER, PAIN)	0.64	1.31	1.42	1.80	1.34	1.67	0.83	0.73	80	0.47	–
36	Interpersonal problems (IMPAIRMENT, SCRL)	1.00	1.44	2.91	2.48	2.98	2.16	0.69	−0.45	128	0.65	–
37	Work &/or school problems (IMPAIRMENT, WRK)	0.96	1.53	3.10	2.32	3.25	2.10	0.84	−1.28	113	0.20	–
38	Alcohol/substance abuse problems (OTHER, ALC)	0.63	1.33	2.35	2.41	2.41	2.12	0.81	0.34	69	0.73	–
39	Lost time (DISSOC, TIME)	0.37	0.89	1.91	2.01	2.41	2.11	0.73	−2.55	58	0.01	0.33
40	Hearing voices inside your head (DISSOC, VOIC)	0.11	0.67	1.42	2.97	0.72	1.34	0.22	0.81	11	0.44	–

Note: *r*_CE_ is the correlation between a symptom *mean Causal association* rating (C) and its respective *mean Effect association* rating (E). *t*_CE_ is the *t*-statistic for the mean difference between a symptom *mean Causal association* rating (C) and its respective *mean Effect association* rating (E); the *df* (*df*_CE_), *p*-value (*p*_CE_), and effect size (*d; d*_CE_) all apply to this mean difference. *d*_CE_ is reported only for statistically-significant differences. All *p*-values apply to two-tailed tests. The Bonferroni-corrected *p-critical for α* of 0.05=0.05/40=0.00125; the corresponding obtained Holm–Bonferroni (Sequential-Bonferroni) threshold was 0.00161.

ANX=anxiety; PTSD=posttraumatic stress disorder; MDD=major depressive disorder; DISSOC=dissociation.

### Reexperiencing of traumatic memories as a cause of depression

Intrusive reexperiencing of traumatic memories (REEXP) was attributed as a greater cause of depression symptoms (i.e., PCR_REEXP→DEP_; M=2.70, SD=2.58) than were depression symptoms attributed as causes of reexperiencing (i.e., PCR_DEP→REEXP_; M=1.92, SD=2.00), *t*(112)=4.95, *p <* 0.001, *d*=0.47. As hypothesized, a significant moderation of the prediction of DEP_FREQ_ by REEXP_FREQ_ was observed for PCR_REEXP→DEP_: ▵*R* ^2^=0.06, *F*(1,109)=10.52, *p=* 0.002. Figure 2 (top) illustrates the simple slopes observed at one SD above and below the mean for REEXP_FREQ_ and PCR_REEXP→DEP_. A similar pattern was observed as for the analysis of ANX_FREQ_ and PCR_ANX→DEP_. As expected, REEXP_FREQ_ predicted DEP_FREQ_ stronger at higher (e.g., one SD above the mean, *b*=0.80 [SE=0.11], *t*[109]=7.46, *p*<0.001) than lower (e.g., one SD below the mean, *b*=0.31 [SE=0.13], *t*[109]=2.48, *p*=0.01) levels of PCR_REEXP→DEP_ scores. Referring to participants at least one SD above the mean for REEXP_FREQ_, the between group difference between those participants who were also one SD above versus below the mean on PCR_REEXP→DEP_ has a large effect size of *d=* 1.26.

In comparison, PCR_REEXP→DEP_ failed to significantly increment in the prediction of DEP_FREQ_ beyond REEXP_FREQ_, ▵*R* ^2^=0.01, *F*(1,110)=1.23, *p*=0.27, ns. Moreover, PCR_REEXP→DEP_ did not significantly mediate the effect of REEXP_FREQ_ in predicting DEP_FREQ_ (*b*=0.022 [SE=0.026], Bonferroni-corrected 95% CI −0.026 to 0.122, ns). The ratio of the indirect to direct effect was 0.036 (SE=0.045), Bonferroni-corrected 95% CI −0.032 to 0.189, ns.

### Anxiety as a cause of depression

Anxiety (ANX) symptoms were attributed as greater causes of depression (DEP) symptoms (i.e., PCR_ANX→DEP_; M=3.23, SD=2.17) than were DEP symptoms attributed as causes of ANX symptoms (i.e., PCR_DEP→ANX_; M=2.33, SD=2.16), *t*(195)=6.74, *p <* 0.001, *d′*=0.48. As hypothesized, the concurrent prediction of DEP_FREQ_ by ANX_FREQ_ was significantly moderated by PCR_ANX→DEP_ (▵*R* ^2^=0.02, *F*(1,192)=7.77, *p <* 0.0167 [*p=* 0.0059]). Figure 2 (bottom) illustrates the simple slopes observed at one SD above and below the mean for ANX_FREQ_ and PCR_ANX→DEP_. As can be seen, ANX_FREQ_ predicted DEP_FREQ_ stronger at higher (e.g., one SD above the mean, *b*=0.19 [SE=0.02], *t*[192]=10.83, *p*<0.001) than lower (e.g., one *SD* below the mean, *b*=0.10 [SE=0.03], *t*[192]=3.79, *p*<0.001) levels of PCR_ANX→DEP_ scores. Referring to participants at least one SD above the mean for ANX_FREQ_, the between group difference between participants also one SD above versus below the mean on PCR_ANX→DEP_ has a large effect size of *d=* 1.54.

In comparison, the alternate hypothesis that PCR_ANX→DEP_ would significantly increment in the concurrent prediction of DEP_FREQ_ beyond ANX_FREQ_ was not supported; ▵*R* ^2^=0.01, *F*(1,193)=3.68, *p*=0.057, ns). Moreover, the alternate hypothesis that PCR_ANX→DEP_ would mediate the effect of ANX_FREQ_ in concurrently predicting DEP_FREQ_ was also not supported (*b*=0.005 [SE=0.004], Bonferroni-corrected 95% CI −0.001 to 0.016, ns). The ratio of the indirect to direct effect was 0.032 (SE=0.021), Bonferroni-corrected 95% CI −0.005 to 0.106, ns.

### Moderated mediation: reexperiencing, guilt–shame and depression

We found that experiences of guilt–shame (SHAME) were attributed as greater causes of depression symptoms (i.e., PCR_SHAME→DEP_; M=3.35, SD=2.60) than depression symptoms attributed as causes of guilt–shame (i.e., PCR_DEP→SHAME_; M=2.65, SD=2.18), *t*(137)=3.78, *p <* 0.001, *d′*=0.32. SHAME_FREQ_ was also correlated with both DEP_FREQ_, *r*(137)=0.15, *p <* 0.05, and REEXP_FREQ_, *r*(279)=0.37, *p <* 0.001. A simple mediation model showed that the concurrent prediction of DEP_FREQ_ from SHAME_FREQ_ (direct effect) and REEXP_FREQ_ (indirect effect) was significant, *R* ^2^=0.53, *F*(2,277)=159.13, *p <* 0.001. In this test, REEXP_FREQ_ partly mediated the association between SHAME_FREQ_ and DEP_FREQ_, *t*(278)=2.66, *p <* 0.05, explaining approximately 14% of the total effect of SHAME_FREQ_ on DEP_FREQ_.

**Fig. 3:**
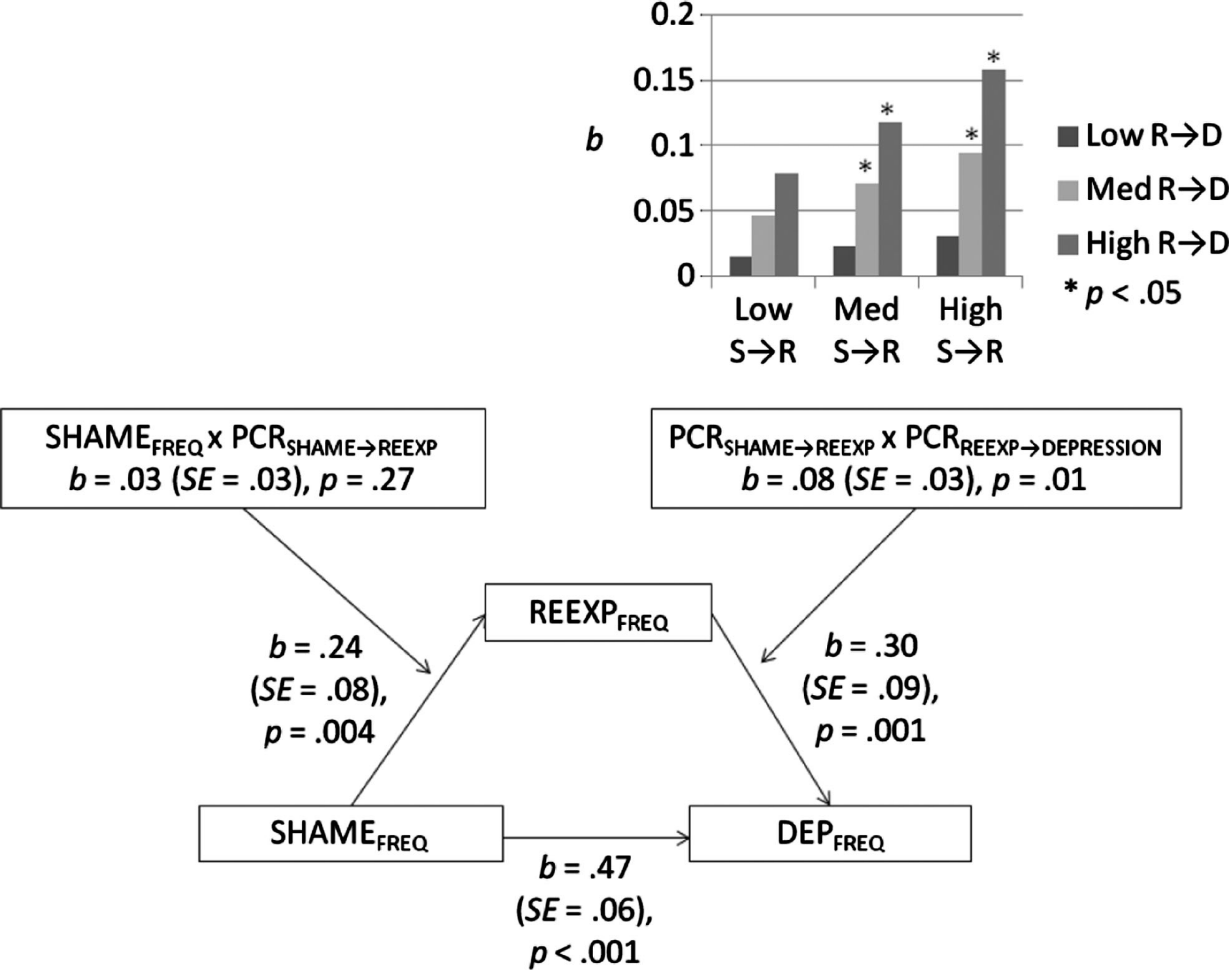
Reexperiencing as a mediator of the association between guilt–shame and depression symptom frequency as moderated by PCR scores.

Figure 3 illustrates the results of the associated moderated mediation model, which examined whether the significance of paths associating SHAME_FREQ_, REEXP_FREQ_, and DEP_FREQ_ was moderated by respective PCR scores. The regression model predicting DEP_FREQ_ from SHAME_FREQ_ (direct effect) and REEXP_FREQ_ (indirect effect) was again significant, *R* ^2^=0.64, *F*(4,66)=29.07, *p <* 0.001. SHAME_FREQ_ was a significant predictor, *b=* 0.471 (SE=0.062), *t*(66)=7.65, *p <* 0.001, as was REEXP_FREQ_, *b=* 0.296 (SE=0.089), *t*(66)=3.34, *p=* 0.001. Critically, the effect of REEXP_FREQ_ in mediating the effect of SHAME_FREQ_ on DEP_FREQ_ was moderated by an interaction between PCR_SHAME→REEXP_ and PCR_REEXP→DEP_, *b=* 0.084 (SE=0.033), *t*(66)=2.52, *p=* 0.01. *Post-ho*c comparisons showed that REEXP_FREQ_ partially mediated the effect of SHAME_FREQ_ on DEP_FREQ_, but only when PCR_SHAME→REEXP_ and PCR_REEXP→DEP_ scores were both at the median or higher (see Fig. 3 top right, bar graph indicating statistical significance of associated beta-weights).

### Mean causal versus effect association scores and causal network of symptoms

**Fig. 4:**
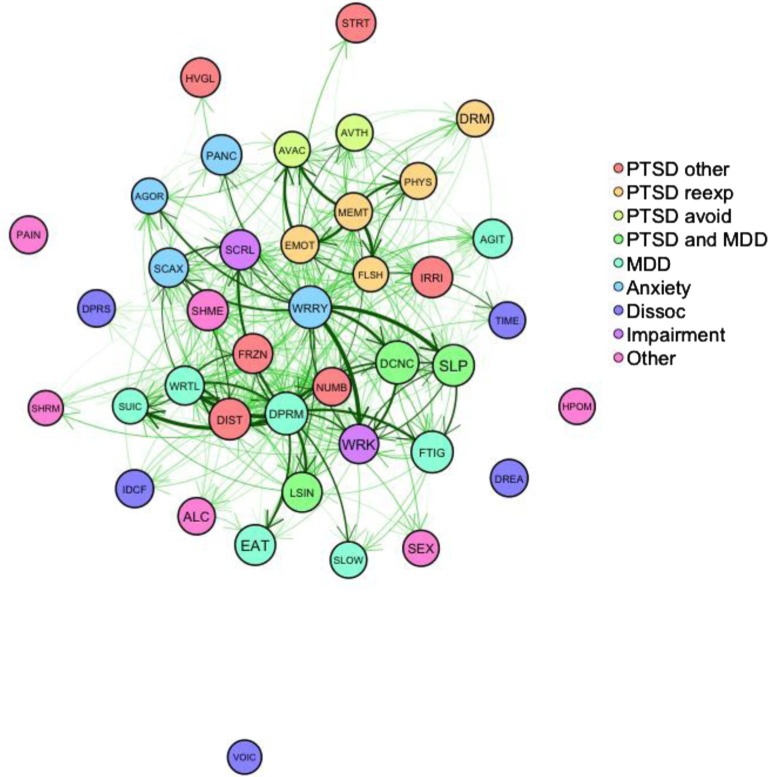
Weighted directed network of mean causal associations between symptoms, averaged over participants who endorsed each two symptoms. Nodes represent symptoms; node color refers to symptom categories. The higher the symptom frequency, the larger the node. Causal associations above three are represented as edges. Moderate causal associations between 3 and 4.5 are shown as thin light transparent edges. Causal associations above 4.5 are shown as darker edges. The thicker and darker these edges, the stronger the association. More strongly connected sets of nodes cluster closer together. For instance, strong direct and indirect connections between “emotional upset at RTE,” “intrusive memories of a TE,” “avoidance of reminders of TE,” and “flashbacks of a TE” leading these nodes to cluster together. Indirect connections can be easily identified, for instance, the association of anxiety and depression can be attributed to the indirect connection from “worrying” via “sleeping problems” to “energy loss/fatigue,” next to the strong direct connections from “anxious worrying” to “sleeping problems” and “difficulty concentrating.”

**Fig. 5:**
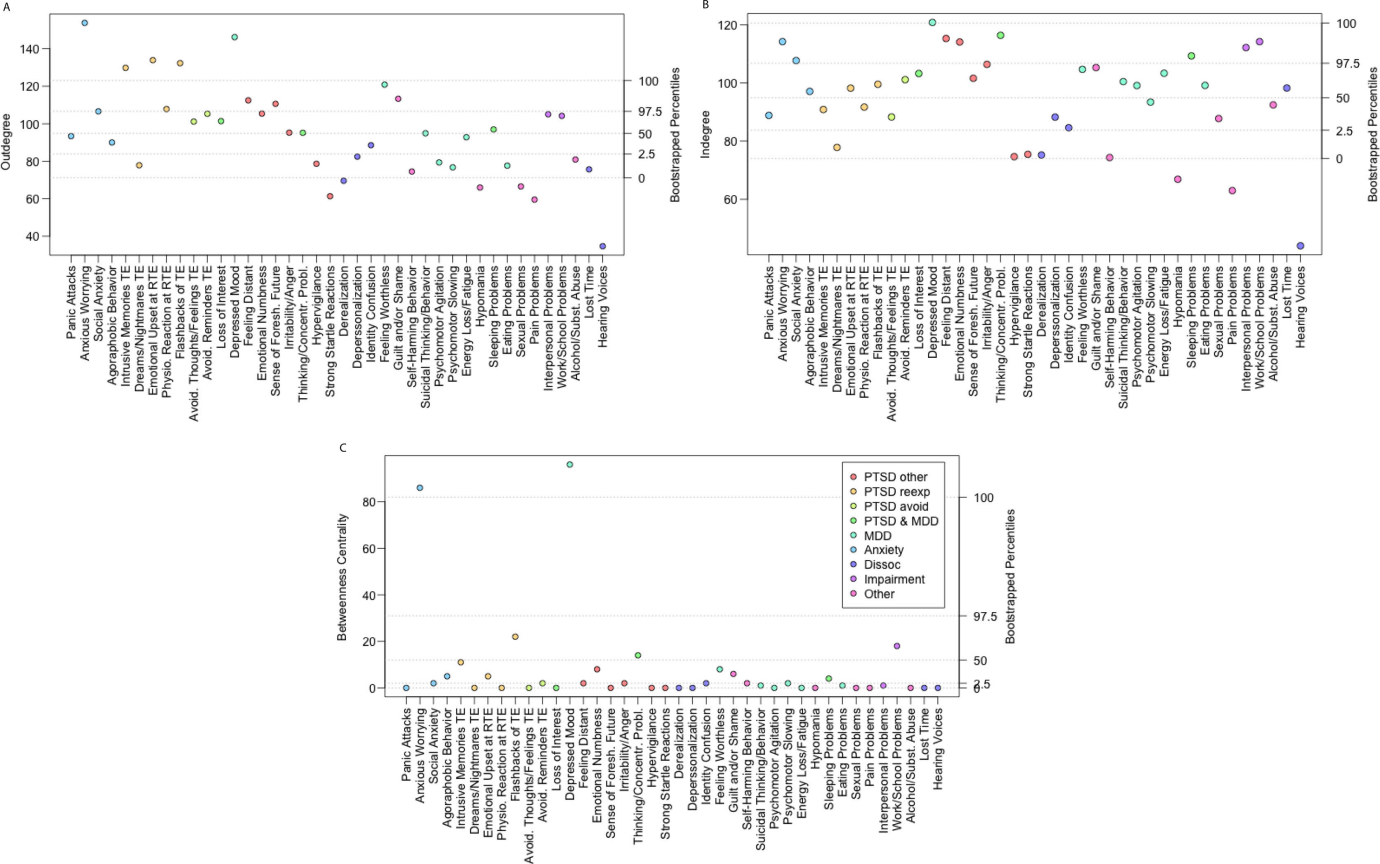
Outdegree (5A), indegree (5B), and betweenness centrality (5C) of each symptom. Symptom categories are color-coded. Observed centrality values are indicated on the left-hand axis and percentiles of interest of the bootstrapped centrality measures on the right-hand axis. For instance, the outdegree (5A) of symptom “emotional upset at reminder of a traumatic event (TE)” is extremely large, even outside the range of the bootstrapped values; the indegree (5B) of symptom “thinking/concentration problems” is extremely large, falling outside the 95% central bootstrapped interval; and the betweenness centrality (5C) of symptom “flashbacks of a TE” is above average, though inside the 95% central bootstrapped interval.

Table 1 indicates the significance of paired differences between mean causal association and mean effect association scores for each of the 40 individual symptoms assessed; the majority of comparisons replicate previous findings (Frewen et al., 2012). Figure 4 represents the network of mean PCR between symptoms, showing clusters of strongly connected symptoms. Figure 5 allows the identification of symptoms with extreme centrality values (outside the 95% central bootstrapped intervals), such as three PTSD symptoms with extremely large outdegrees (Fig. 5A), a different set of PTSD symptoms with large indegrees (Fig. 5B), and the symptoms “Depressed mood” and “Anxious worrying,” which exhibited extremely large betweenness centrality (Fig. 5C).

**Fig. 6:**
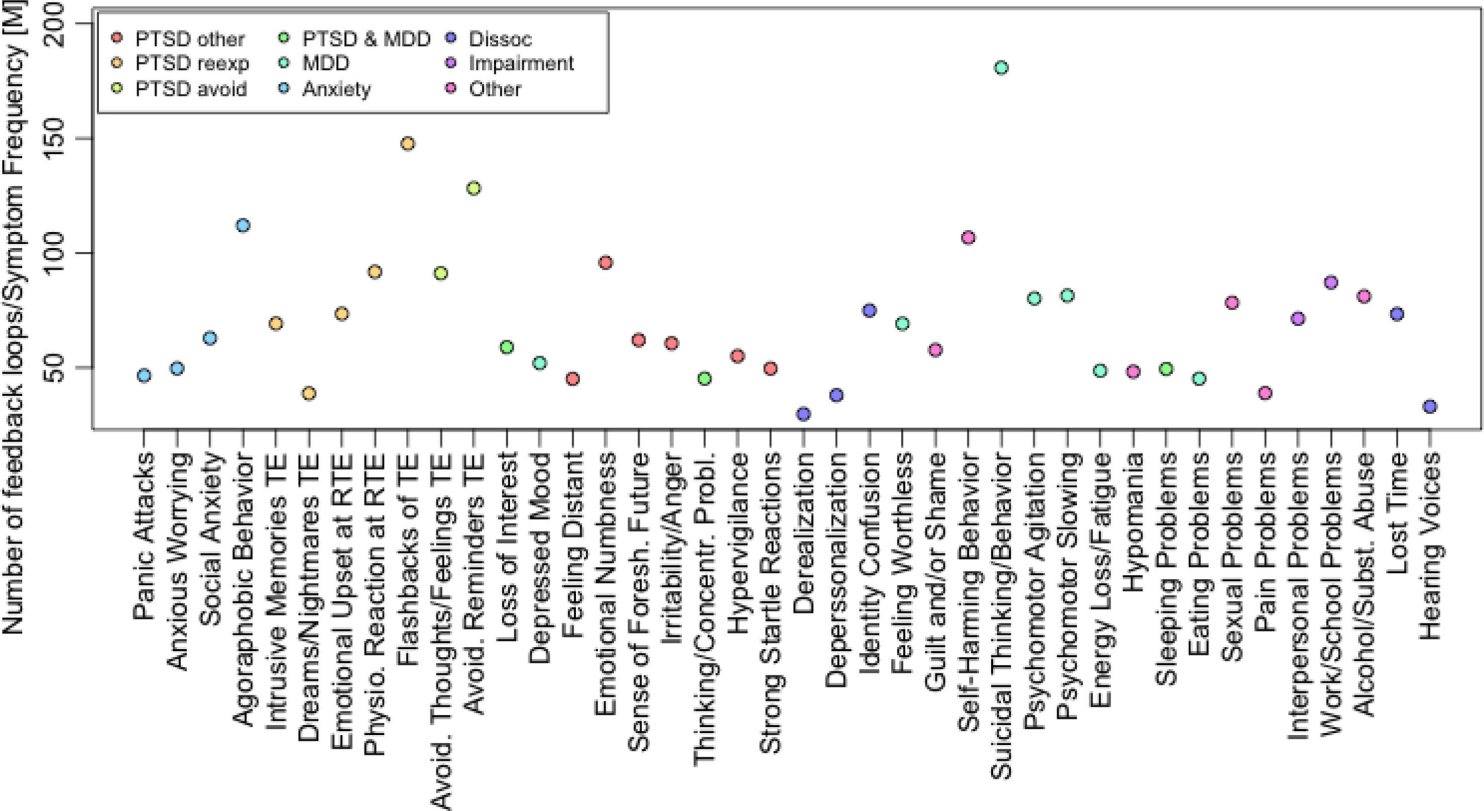
Number of feedback loops, in which a symptom is involved, corrected for symptom frequencies. Symptom categories are color coded.

The analysis of feedback loops in each participant's network of PCR scores revealed 281,936 unique feedback loops. Participants’ number of feedback loops ranged from 0 to 98,477. Several PTSD symptoms figure dominantly in feedback loops, among other symptoms, as shown in Fig. 6. Among PTSD symptoms, “Flashbacks of TE” and “Avoidance of reminders of TE” were involved in feedback loops most often, whereas “Dreams/nightmares of TE” were minimally involved. As predicted, Spearman's rho revealed a statistically significant relationship between the number of feedback loops and the symptom frequency sum score (*r*[288]=0.67, *p*<0.0001). This relationship remained significant after partialling out the number of symptoms (*r*[288]=0.23, *p*<0.0001), and both the number of symptoms and the number of PCR scores (*r*[288]=0.23, *p*<0.0001).

## Discussion

This study further examined PCR *scaling* as an assessment methodology for measuring participants’ own attributions concerning the direction and magnitude of possible cause-and-effect associations between their presenting problems. We investigated associations between reexperiencing of traumatic memories, depressive symptoms, anxiety, and guilt–shame, in addition to related psychological problems within complex, multi-symptom networks.

Support was found for our moderation models of the association between PCR scores and symptom frequency scores (Fig. 1A and 1B). In contrast, the alternate hypotheses, those conceptualizing PCR scores as incremental predictors (Fig. 1C) or mediators (Fig. 1D) of the association between symptom frequencies, failed to reach corrected levels of significance. Supporting PCR scaling as a participant-specified approximation of the directional strength of associations between the frequency of their psychological symptoms, moderation analyses showed that the extent to which participants’ reexperiencing and anxiety symptom frequencies concurrently predicted their depressive symptom frequency varied with the magnitude with which participants themselves perceived their reexperiencing and anxiety symptoms to be a cause of their depression (Fig. 2). Moreover, results showed that the effect of reexperiencing symptom frequencies, hypothesized and found to be a partial mediator of the relationship between guilt–shame and depression symptom frequencies, was further moderated by the degree to which participants’ attributed their guilt–shame symptoms as a cause of their reexperiencing symptoms and, in turn, attributed their reexperiencing symptoms as a cause of their depression (Fig. 3). Accordingly, our moderation results indicate that, if one wants to know how depressed an individual is *by means* of knowing how bothered she is by memories of past traumatic events, and/or how anxious she is, one might ask how much she regards her intrusive recollections and anxiety symptoms to be significant causes of her depression (i.e., via PCR scaling). The results of our moderation models give some support to interpreting PCR scaling as a participant-specified approximation of the predictive strength associating different clinical problems. As such, PCR assessments may have clinical utility in providing a psychometric method for directly assessing whether two or more presenting problems are interrelated, if only as perceived within the person experiencing them. The perceived interrelated nature of many psychological symptoms, as revealed by PCR scaling, also theoretically supports intrinsic interactions between symptoms as an explanation of symptom clustering into syndromes and of comorbidity between psychological disorders (Borsboom, 2008; Borsboom & Cramer, 2013; Cramer et al., 2010).

Mediation and moderation analyses were carried out using mean PCR scores that were aggregated at the level of disorders. In future research, network techniques should be developed to assess similar effects at the level of individual symptoms. Fitting a model that utilizes the topology of the network organization to the symptom data could carry this out. Results of the network analysis, in particular, the analysis of feedback loops, suggest that more mediation and moderation effects are likely to be present on the level of individual symptoms. This feedback loop analysis of the causal networks on the idiographic level revealed large differences in the involvement of different PTSD symptoms in feedback loops. Flashbacks of traumatic events and the avoidance of reminders of traumatic events were found in large numbers of feedback loops, whereas nightmares of traumatic episodes were a less frequent element in feedback loops. In addition, as hypothesized, the number of feedback loops in a network was positively related with symptom frequency scores, suggesting coherence of the perceived causal structures with symptom frequencies.

Network analyses identified different sets of symptoms that were perceived as influencing other symptoms (outdegree), being influenced by other symptoms (indegree), and/or transmitting between different symptoms (betweenness centrality). While anxious worrying, depressed mood, and remembering or being reminded of traumatic events were reported as strongly influencing other symptoms, the symptoms reported as most influenced by other symptoms included those describing internal emotional states (e.g., depressed mood, emotional numbness) or problems with work, school, social interactions, concentration, and sleeping. A substantial transmitting role was also attributed to anxious worrying and depressed mood. Consistent with the hypothesis that comorbidity arises due to causal relations between symptoms (e.g., Borsboom, 2008; Borsboom & Cramer, 2013; Borsboom et al., 2011; Cramer et al., 2010; Schmittmann et al., 2011), PCR between symptoms of different disorders were reported in the present sample.

Limitations of the present research should be acknowledged. Sample sizes were small particularly for analyses of PCR ratings incorporating multiple variables; although statistical significance was observed, reliability and type II errors are potential concerns. All data was collected exclusively by self-report, and symptom assessments did not use standardized measures. Additionally, we did not include a measure of trauma exposure such that intrusive reexperiencing symptoms may have related to stressful yet non-“traumatic” events as typically defined by psychotraumatologists, granting that generally stressful but non-traumatic events are also the frequent cause of intrusive reexperiencing (e.g., review by Brewin et al., 2010). As a result, the clinical significance of the symptomatology experienced by the current sample is uncertain, particularly given that data was collected from a university student sample rather than participants with diagnosed psychiatric conditions. Therefore, findings concerning PCR scaling identified herein may not generalize to patient samples, and studies of clinical samples are needed. Future studies of PCR involving shame and guilt should examine the roles of these concepts distinctly rather than in a single item, in accordance with current theory of depression (e.g., Kim et al., 2011) and PTSD (e.g., Lee et al., 2001; Wilson et al., 2006).

The question, to what extent participant's causal attributions overlap with actual causal relations, is beyond the scope of this study. Such a study is complicated, because actual causal relations are difficult to establish. Based on our findings, we recommend an intensive high-frequency longitudinal study to infer an individual's actual causal model, along with PCR measurements from the participant and another person (e.g., partner and/or therapist; Frewen et al., 2012). A comparison of perceived and actual causal relations would seem to offer several possibilities. For instance, a participant with poor insight into their actual causal relations between experienced symptoms might benefit from a different treatment program than someone with excellent insight, in particular where behavioral adaptations of an individual maintain or aggravate the disorder (e.g., avoidance of anxiety triggers).

While acknowledging limitations of this study, we further offer PCR scaling as a psychometric assessment framework that may be worthy of evaluation in clinical samples as an aid to case conceptualization. Specifically, PCR scaling provides a systematic psychometric approach to evaluating “*what goes with what*” amid the myriad clinical symptoms often presented by individuals with complex mental health problems and trauma histories, at least as conceived by the individual her or himself. We argue that such attributions are worthy of assessment in and of their own right. The degree to which participants’ attributions about causality among their presenting problems match actual causal associations is a matter for further research.

## Footnotes

1 To illustrate the difference, consider two hypothetical symptoms. Say, symptom A has been reported to cause merely one other symptom with average score 3, and symptom B causes four other symptoms with average scores of 3, 2, 4, and 3. Now, while the mean causal association scores of symptoms A and B both equal 3, the outdegree of symptom A would equal 3, and the outdegree of symptom B equals 3+2+4+3=12. This holds analogously for indegree and mean effect association scores.
